# Exercise interveNtion outdoor proJect in the cOmmunitY for older people – results from the ENJOY Seniors Exercise Park project translation research in the community

**DOI:** 10.1186/s12877-020-01824-0

**Published:** 2020-11-04

**Authors:** Pazit Levinger, Maya Panisset, Jeremy Dunn, Terry Haines, Briony Dow, Frances Batchelor, Stuart Biddle, Gustavo Duque, Keith D. Hill

**Affiliations:** 1 National Ageing Research Institute, Melbourne, Australia; 2 Institute for Health and Sport, Victoria University, Melbourne, Australia; 3 Rehabilitation, Ageing and Independent Living Research Centre, Monash University, Melbourne, Australia; 4 School of Primary and Allied Health Care, Monash University, Melbourne, Australia; 5 Centre for Health Policy, University of Melbourne, Melbourne, Australia; 6 School of Nursing and Midwifery, Deakin University, Melbourne, Australia; 7 Department of Physiotherapy, The University of Melbourne, Melbourne, Australia; 8 Centre for Health Research, University of Southern Queensland, Brisbane, Australia; 9 Australian Institute for Musculoskeletal Science (AIMSS), Melbourne Medical School, Melbourne University, Melbourne, Australia

**Keywords:** Physical activity, Older people, Falls prevention, Seniors Exercise Park, Outdoor

## Abstract

**Background:**

Many research studies evaluate physical activity interventions for older people in the community, however relatively few successfully promote maintenance of physical activity beyond the completion of the intervention. This study aimed to implement and evaluate the effects of sustained engagement in physical activity on mental, social and physical health outcomes through the use of the Seniors Exercise Park physical activity program for older people (the ENJOY project).

**Method:**

People aged ≥60 years underwent a 12-week structured supervised physical activity program using outdoor exercise park equipment followed by 6 months unstructured independent use of the exercise park. Participants were assessed at baseline, 3 months and 9 months and completed a test battery evaluating physical activity, physical function and health related quality of life measures. Repeated measures ANOVA was used to compare differences between baseline, 3 and 9 months.

**Results:**

Of the 95 participants, 80 (84.2%) completed the 3 months supervised program, and 58 (61%) completed the 9 month assessment (the latter impacted by COVID-19 restrictions). A significant increase in physical activity level was demonstrated following the 12 weeks intervention (*p *< 0.01). Significant improvements were also demonstrated in all physical function measures (*p* < 0.01), self-rated quality of life (*p *< 0.05), wellbeing (*p *< 0.01), fear of falls (*p *< 0.01), falls risk (*p *< 0.01), depressive symptoms (*p* = 0.01) and loneliness (*p* = 0.03) at 3 months. At the 9 months follow up, significant improvements from baseline were demonstrated in the frequency, duration and total of physical activity level (*p *< 0.05), and all physical function measures (*p *< 0.05), with no decline in these measures from 3 to 9 months. At 9 months, significant changes were observed in the health related quality of life mobility and self care domains with reductions in both fear of falls and falls risk (*p <* 0.05).

**Conclusion:**

The Seniors Exercise Park may be an effective modality for improving and sustaining older people’s physical function and wellbeing and can be an important public health infrastructure investment in promoting physical activity for older people. Future work should focus on wider implementation of the program and on scaling up this initiative to achieve public health benefit for the community.

**Trial registration:**

Trial registration number ACTRN12618001727235, Date of registration 19th October 2018, https://www.anzctr.org.au/Trial/Registration/TrialReview.aspx?id=375979

## Background

The world’s population is ageing rapidly, with the number of older people age 65 and over projected to more than double by 2050 [1]. The number of Australians aged 65 and over is expected to increase from around 2.5 million in 2002 to 6.2 million in 2042 [2]. Physical activity is one of the key behavioral factors to positively impact health outcomes, including reduction of risk of chronic diseases, cognitive and functional decline, and improvement in mental health [3]. Increase in physical activity can also minimise the burden on the health care system [4]. Despite the strong evidence around the importance of physical activity, older people do not regularly undertake physical activity [5], with less than 25% of older Australians meeting the recommended physical activity guidelines [6].

There has been considerable research into physical activity interventions for older people in the community, but interventions that successfully promote maintenance of physical activity beyond the completion of the intervention are limited [7, 8]. Various methodological challenges exist that often limit translation of physical activity programs into practice, these include: lack of evidence of transferability of trial results to the community setting, insufficient local expertise to roll out community exercise programs, and inadequate infrastructure to integrate evidence based programs into community practice [9]. Interventions that are designed to be conducted in a community setting with community engagement have the potential to be sustained beyond the trial period and have shown to be effective in increasing and promoting physical activity [10, 11].

In recent years, outdoor environments and associated infrastructure features (e.g., exercise equipment) have been recognized as an important investment to promote regular physical activity [12, 13]. Hence, the design of an age friendly ‘active environment’ has been recommended as one of the strategies to increase physical activity at a population level [14]. In 2012 we commenced our research work in the area of age friendly active spaces for older people with the utilization of outdoor exercise equipment specifically designed for older people (the Seniors Exercise Park). The Seniors Exercise Park program was designed to actively promote community well-being through the provision of a unique exercise and social support program. In a small 18 week randomized controlled trial (RCT), we demonstrated the effectiveness of the Seniors Exercise Park program on improving physical function and social health in older people [15, 16]. These preliminary positive outcomes indicated the need for investigation of its sustained impact on physical and social health outcomes, and its potential wider usage in the community on a larger scale with local governments’ (councils’) engagement. Therefore, the aim of the present study was to implement and evaluate the effects of sustained engagement in physical activity on physical, mental, social and health outcomes through the use of the Seniors Exercise Park physical activity program for older people (the ENJOY project).

## Methods

### Design and setting

**Fig. 1:**
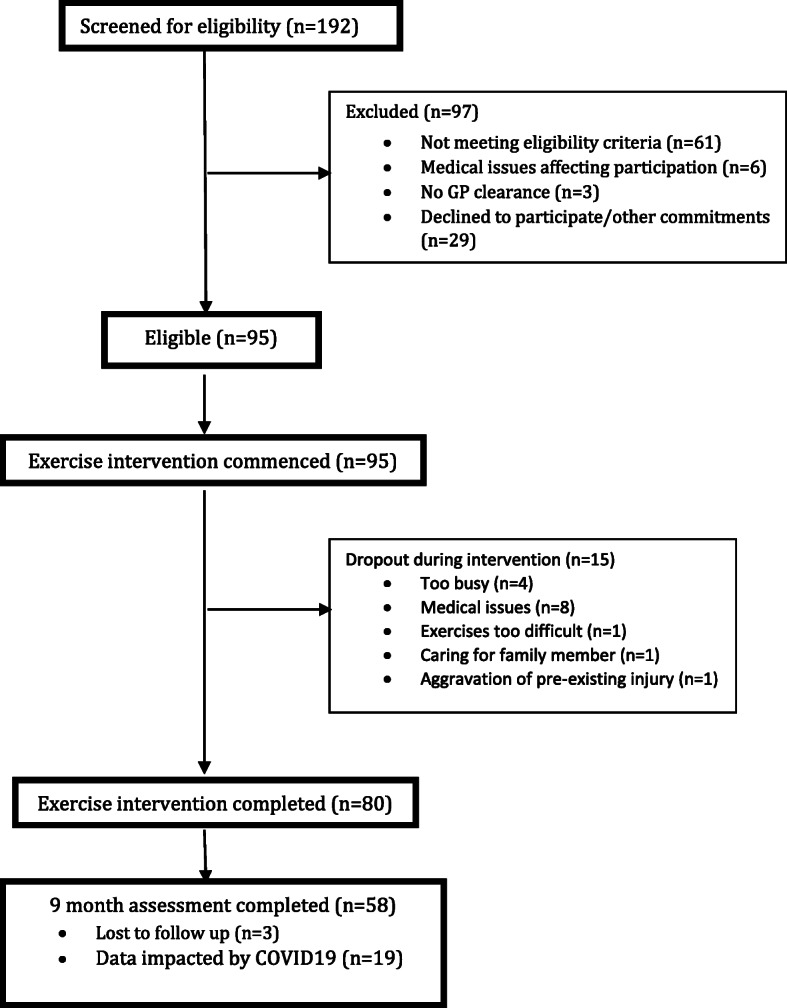
Flow diagram of recruitment and drop out

This study was a multi-site prospective study with a pre and post intervention design and 9 month follow up. Participants underwent a 12-week structured supervised physical activity program using outdoor exercise park equipment followed by a 6 month unstructured physical activity program, including ongoing unsupervised access to the exercise park. Each exercise session was followed by a social gathering with morning/afternoon tea provided by the research team. Participants were assessed at baseline, post intervention (3 months) and 9 months follow up time points as detailed in Fig. 1. The study was designed according to the Transparent Reporting of Evaluations with Nonrandomized Designs (TREND) [17] which complements the widely adopted Consolidated Standards Of Reporting Trials (CONSORT) statement developed for randomized controlled trials [18]. Ethical approval was obtained from the Melbourne Health Human Research Ethics Committee, Melbourne (Application ID. HREC/18/MH/286, local number 2018.238). All participants provided informed consent. The full description of the study’s methods, design, and procedure can be found in the trial protocol [19].

### Study population

#### Inclusion criteria

Older people were eligible to participate in the study if they:

1) were aged 60 years and over living in the community (i.e. not living in an institution, such as a nursing home); 2) had one or more falls in the previous 12 months or were concerned about having a fall; 3) were generally independent around the house (able to take care of themselves) and in the community (e.g. able to walk away from home to visit local stores, friends, and other local venues), and able to attend the outdoor exercise park; 4) were able to walk outdoors and use the exercise equipment with no more gait aid support than a single point stick 5) did not have cognitive impairment (Abbreviated Mental Test Score (AMTS) > 7/10) [20].

#### Exclusion criteria

Older adults were excluded from this study if they: 1) had neurological or musculoskeletal conditions limiting walking to less than one block; 2) had a history of stroke, Parkinson’s disease, or other neurological disorder impacting on mobility; 3) were unable to understand conversational English; 4) were taking part in a structured resistance training and/or an organised balance training program more than once a week; 5) met the Australian physical activity recommendations of 150 min of physical activity / week [21]; 6) had any documented medical condition or physical impairment that was deemed by their medical practitioner to contraindicate their inclusion.

### Recruitment

Older people were recruited from the general community in the suburbs close to the Seniors Exercise Parks location in Melbourne, Australia. Advertisements in local newspapers, council newsletters, posters displayed on notice boards, and flyers distributed to senior groups were used for recruitment. Information was also placed online on the councils’ and participating partners’ websites as well as associated social media platforms (e.g. Facebook, Twitter). Recruitment took place between October 2018 to November 2019.

### Procedure

Participants who met the inclusion criteria attended an initial (baseline) assessment at a community centre close to their area of residence. Demographic characteristics (age, gender), anthropometric measures (height and weight), previous medical history, current medication usage, socioeconomic and cultural background information (e.g. employment, level of education, country of birth, years of residency in Australia) and falls history were collected at baseline. Assessments were undertaken at baseline, 3 months and 9 months, by an allied health professional (Accredited Exercise Physiologist and/or Physiotherapist).

### Assessments

#### Primary outcome

##### Physical activity

The level of physical activity of the participants was measured using the Community Healthy Activities Model Program for Seniors (CHAMPS) [22]. The CHAMPS provides a self-reported measure of caloric expenditure (and frequency) per week in all exercise-related activities and caloric expenditure (and frequency) per week in moderate exercise -related activities.

#### Secondary outcomes

A comprehensive suite of physical function (strength, balance, functional mobility), psychosocial (quality of life, enjoyment, social isolation, fear of falls, loneliness), and mental health outcomes (mental wellbeing, depression) and falls risk assessment were undertaken as detailed in the protocol paper [19], and summarized below.

##### Physical function measures

Physical measures of strength, balance and functional mobility were assessed using the following validated tests.

(i) Functional lower limb muscle strength was assessed using the 30-s sit to stand test [23]; (ii) Exercise tolerance and functional mobility was assessed using the two-minute walk test [24]; (iii) Dynamic balance was assessed using the step test [25], the sum of the number of steps from each limb was combined and used for the analysis [26]; and (iv) Walking speed was assessed using the 4 m walk test [27].

##### Psychosocial, mental and quality of life health outcomes

Psychosocial, mental health and quality of life outcomes were assessed using the following questionnaires:

(i) Health-related quality of life was assessed using the EQ-5D-5L [28]. The EQ-5D-5L comprises five dimensions (mobility, self-care, usual activities, pain/discomfort and anxiety/depression) as well as an overall self-rated health status (Visual Analog Scale (VAS) 0–100) where higher score represents better health.
(ii) *Mental wellbeing* was assessed using the five-item World Health Organization (WHO-5) Wellbeing questionnaire [29, 30]. The WHO-5 measures psychological wellbeing and depressive symptoms using 5 simple questions. The raw score was calculated to obtain a percentage score, which ranges from 0 representing the worst imaginable wellbeing and 100 representing the best imaginable well-being.
(iii) *Loneliness* was assessed using the UCLA 3-item Loneliness Scale which incorporates three dimensions of loneliness: relational connectedness, social connectedness and self-perceived isolation [31, 32]. The scale gives a possible range of scores from 3 to 9 (higher scores indicate greater feelings of loneliness).
(iv) *Depression* was assessed using the short version Geriatric Depression Scale (GDS-15) where a score of 0 to 5 is considered normal and a score greater than 5 suggests depressive symptoms [33].
(v) *Fear of falls* was assessed using The Short Falls Efficacy Scale International (Short FES-I) questionnaire [34], a 7-item scale ranging from 7 (no concern about falling) to a maximum 28 (severe concern about falling).
(vi) *Self-efficacy* barriers to exercise was assessed using The Self-Efficacy for Exercise (SEE) questionnaire, with scores ranging from 0 to 90 (a higher score indicates higher self-efficacy for exercise) [35].
(vii) *Enjoyment* was assessed using the 8-item version Physical Activity Enjoyment Scale (PACES), where higher values reflect greater levels of enjoyment (values range 8–56) [36].
(viii) *Social isolation and social support* were assessed using the short version 6-item Lubben Social Network Scale (LSNS6). The score ranges between 0 and 30 where higher scores indicate more social engagement [37].

##### Falls risk assessment

(i) *The Falls Risk for Older People in the Community (FROP-Com)* risk assessment tool was used to assess fall risk. The FROP-Com consists of 13 falls risk factor domains, with most risk factors scored to reflect graded risk on a 4-point scale (nil, mild, moderate, or severe) [38].

### Exercise park Intervention

#### The Seniors Exercise Park

**Fig. 2:**
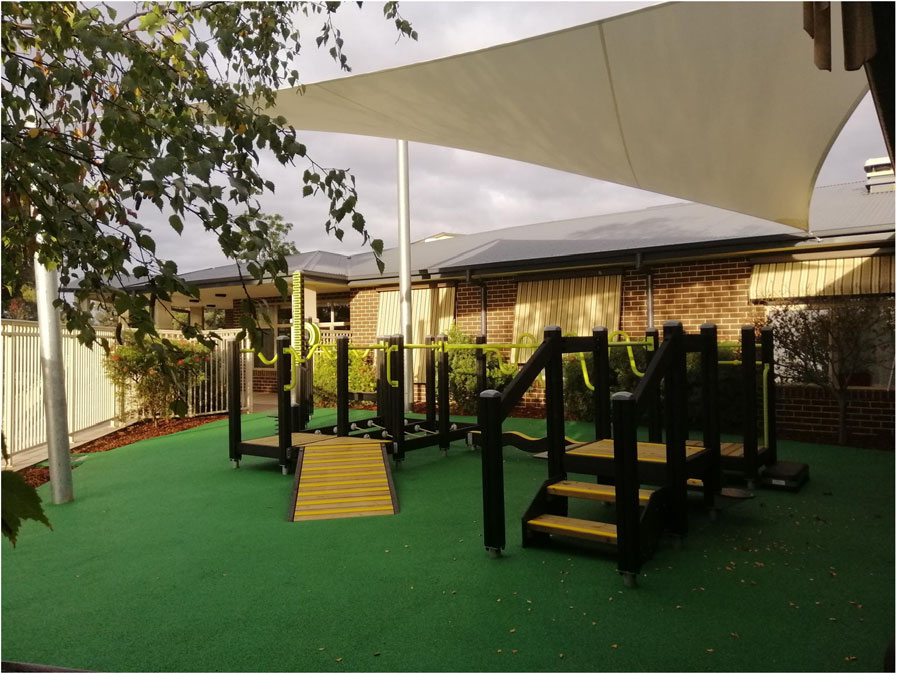
The Seniors Exercise Park at Leith Park, St Helena

The Seniors Exercise Park equipment (Lark Industries (Australia) and Lappset Group) consists of outdoor playground equipment specifically designed for older people to improve strength, balance, joint movements and overall mobility and function (Fig. 2). It comprises multiple equipment stations that target a specific function or movement (upper and lower limb) such as shoulder range of movement, static and dynamic balance (unstable surfaces), and functional movements (walking up/down stairs, sit to stand). The exercise park equipment was installed in two public locations and a third location in a retirement living and aged care community respectively: Barry Rd. Community Centre, Thomastown, Melbourne (under the municipality of Whittlesea City Council); Central Park Community Centre, Hoppers Crossing, Melbourne (under the municipality of Wyndham City Council); and Leith Park, St Helena, Melbourne (Old Colonists’ Association of Victoria).

#### 12-week structured supervised exercise program

Participants participated in a 12-week supervised exercise intervention program twice a week using the Seniors Exercise Park. The exercise program was delivered by a qualified exercise instructor (Accredited Exercise Physiologist or Physiotherapist). Participants performed exercises that focused on strength, balance, coordination, mobility and flexibility as detailed in our previous work [39]. Each session consisted of 5–7 min of warm-up exercises, followed by 45–75 min at the equipment stations, and concluded with 5 min of cool down exercises (overall duration approximately 80 min). The exercise classes were run as a circuit-based group program with 6–10 participants. Each participant was familiarized with the exercises individually and the difficulty level was tailored to the capabilities of the participant. Each session was followed by morning/afternoon tea to encourage socialization.

##### Individual and group exercise progression

Each exercise station included two different exercises which were performed twice by each participant. Examples of the stations and the exercises can be found at https://youtu.be/PaYuCMtnlYk. Two participants were allocated to each station such that each participant performed one exercise for the allotted time and then swapped with their partner, repeating each exercise twice before rotating to the next station. Rest periods were provided during transition to the next station. The duration of each exercise and rest period were adjusted progressively according to program progression, as detailed in the protocol paper [19].

#### Participation rate (adherence) and exercise monitoring

##### During the 12-week supervised exercise program

Frequency of exercise session participation was determined using daily attendance logs kept by the exercise instructor. Overall adherence to the structured exercise program was defined by the number of sessions attended: where 100% adherence indicated that a participant attended all available 24 sessions. In the event of cancellation (due to weather or public holidays), participants were given alternative make up sessions to achieve the 24 sessions.

##### Monitoring exercise uptake following the 12-week exercise program for 6 months – fob access system

At the completion of the structured 12 weeks exercise program participants were given two options to choose from to continue their physical activity. Option 1 – independent unsupervised access and usage of the exercise park in participants’ own preferred time. Option 2: access to twice a week exercise sessions on the exercise park under supervision but with no formal structured group activity. Adherence and exercise uptake for the 6 months post intervention was monitored using a fob access system (CityWatch Security, Melbourne, Australia www.citywatchsecurity.com.au/) that included a card reader/scanner and a control panel at each site. Participants were assigned an individual identification key (fob) which they used to tap a card reader each time they accessed the Seniors Exercise Park at the site. Their access was recorded and monitored (thereby electronically monitoring access). A separate paper will report outcomes and experiences using the fob system.

#### Safety considerations and adverse events

##### Weather elements

In extreme weather conditions (e.g. heavy rain, extreme heat (above 30 °C)), if deemed by the exercise instructor as unsafe to exercise, sessions were cancelled. In circumstances where sessions were cancelled, or during a holiday period, makeup sessions were organised towards the end of the program (up to two weeks or 4 sessions). Any cancellation and the associated reason were recorded in a log book kept by the researchers.

### Adverse events

#### Joint pain/discomfort and or muscle soreness

Instances of joint pain or discomfort (directly related to the exercise program) during the exercise program were recorded. Sessions that were missed due to pain or discomfort that had not settled and prevented a participant from attending the exercise sessions were also recorded.

#### Falls

Any falls during the delivery of the structured supervised exercise programs and during the independent usage phase of the Seniors Exercise Park were recorded. A fall was defined as an event when the participant ‘inadvertently comes to rest on the ground, floor or other lower level’ (WHO Global Report on Falls Prevention in Older Age [40]).

#### Serious adverse events - cardiorespiratory adverse reaction

Any report of difficulty breathing that did not settle quickly with rest, new or unrelenting chest pain, or acute changes in the level of consciousness during the session were documented. A serious adverse event was deemed if symptoms did not settle and medical emergency care was required and organised.

### Power analysis

A power analysis was undertaken using previously published data using the primary outcome measure CHAMPS for measurement of change in physical activity level over a 9 month period [22]. We considered a minimum meaningful change in the physical activity outcome from use of the Seniors Exercise Park intervention to be d = 0.33. Using this standardized effect size, 90% power and a two-tailed alpha of 0.05, we calculated need for a sample size of 98 participants. Previous data indicates a within subject change in daily calorie expenditure for all activities of 1509 and for moderate intensity activities of 1196 when exposed to 6-month physical activity program [22, 41]. This meant we were likely to have 90% power to detect a change of 503 in total daily calorie expenditure, and 399 in daily calorie expenditure in moderate intensity activities. We projected for a 15% drop-out rate, thus we sought a total sample of 113 participants (37–38 per site).

### Statistical analyses

For the primary outcome of overall physical activity score (CHAMPS) and the physical, mental, and health secondary outcome measures, repeated measures analysis of variance (ANOVA) were used to determine if there were differences between scores collected at baseline assessment and at 9 month follow-up. A separate repeated measures ANOVA (with the equivalent non parametric test for ordinal data) was used to examine the effect of the exercise program on physical activity level, physical, mental and psychosocial and health outcomes between baseline and 3 months; and 3 months vs 9 months. Information collected about exercise adherence were reported using descriptive statistics (% of adherence). Data were analysed using SPSS version 26.0 (IBM Corp, NY, USA). Effect size, Partial Eta Squared, (${\eta}_p^2$) from SPSS was used to determine effect size as follows: ${\eta}_p^2$ values greater than 0.14 were considered a large and significant effect size whereas 0.01 and 0.06 were considered small and medium, respectively [42].

## Results

Ninety-five older people living in the community who volunteered to participate were eligible to take part in the study, with a mean age of 73.0 ± 7.4, and 82.1% female. The majority of participants (94.7%) suffered from at least one medical condition with the most common conditions reported being arthritis (70.1%), hypertension (62.1%) and hypercholesterolemia (51.6%) (Table 1). Fifteen participants dropped out between baseline and three months follow up (15.7%), leaving 80 participants available for analysis of pre-post intervention (mean age 72.8 ± 7.5 years; 81.3% females). No significant differences existed between those who dropped out and the remaining sample with respect to their medical or demographic characteristics. Recruitment and drop out breakdown are provided in Fig. 1. Interruption to data collection occurred during the COVID-19 pandemic due to the physical distancing and lock down restrictions which prevented access to the Seniors Exercise Park. Participants were not able to access the Seniors Exercise Parks for a lengthy period of several months (restrictions of public parks closure and no access to aged care sites as imposed by the Australian State Government). Data of *n* = 19 was impacted due to COVID-19 and were excluded from the analysis (Fig. 1). Consequently, a separate analysis was conducted for the comparison of the CHAMPS primary and secondary outcomes between baseline and 9 months follow up (*n* = 58), in addition to the baseline vs 3 months analyses (*n* = 80).

**Table 1:** Participants’ characteristics

	Overall sample ***n*** = 95	Completed intervention ***n*** = 80	Drop out ***n*** = 15
Age (yrs), mean ± SD	73.0 ± 7.4	72.8 ± 7.5	74.13 ± 7.03
Females (%)	78 (82.1)	65 (81.3)	13 (86.7)
Height (m), mean ± SD	1.6 ± 0.08	1.6 ± 0.9	1.6 ± 0.7
Weight (kg), mean ± SD	78.3 ± 16.8	76.6 ± 15.2	87.1 ± 22.1
BMI (kg/m^2^), mean ± SD	29.9 ± 5.8	29.3 ± 5.4	32.9 ± 7.3
Falls in preceding 12 months (%)	49 (51.5%)	42 (52.5%)	7 (46.6)
Medical conditions and musculoskeletal conditions n (%)			
Arthritis (Osteoarthritis/Rheumatoid Arthritis)	67 (70.5)	55 (68)	12 (80)
Hypertension	59 (62.1)	48 (60)	11 (73.3)
Hypercholesterolemia	49 (51.6)	44 (55)	5 (33.3)
Hearing impairments	43 (45.3)	31 (38.7)	12 (80)
Cardiovascular conditions	28 (29.5)	22 (27.5)	6 (40)
Incontinence	26 (27.4)	20 (25)	6 (40)
Respiratory conditions	24 (25.3)	18 (22.5)	6 (40)
Osteoporosis	21 (22.1)	19 (23.8)	2 (13.3)
Diabetes mellitus	18 (18.9)	15 (18.8)	3 (20)
Other metabolic conditions (Kidney/Thyroid Disorder)	28 (29.4)	20 (25)	8 (53.3)
Medication usage and type, n (%)			
Taking medications	85 (89.4)	71 (90)	14 (93.3)
Median number of medications (Interquartile Range)	6 (5)	6 (6)	6 (4)
Hypertensive medications	59 (62.1)	47 (58.8)	12 (80)
Cholesterol-lowering medications	45 (47.3)	40 (50)	5 (33.3)
Blood Thinners	29 (30.5)	25 (31.3)	4 (26.7)
Pain relieving medications	26 (27.4)	26 (32.5)	2 (13.3)
Anti-depressant medications	21 (22.1)	16 (20)	5 (33.3)
Respiratory medications	19 (20)	16 (20)	3 (20)
Glucose lowering medications	13 (13.7)	12 (15)	2 (13.3)
Anti-inflammatory medications	10 (10.5)	9 (11.3)	1 (6.7)
Socio-economic and education status			
Education level - Secondary school or below (%)	60 (63.1)	50 (62.5)	10 (66)
First Generation migrants (born overseas) (%)	35 (36.8) Europe 20 (57.1); Middle East 6 (17.1); Asia 5 (14.3); America 1 (2.8); South Africa 2 (5.7), Oceania 1 (2.8)	32 (40) Europe 17 (53.1); Middle East 6 (18.8); Asia 5 (15.6); America 2 (3.1); South Africa 1 (6.3), Oceania 1 (3.1)	3 (20) Europe 3 (100)
Second Generation migrants (parents born overseas)	51 (53.6)	44 (55)	7 (46.7)
Speak other language than English	18 (18.9)	16 (20)	2 (13.3)
Marital status			
Married/spouse	50 (52.6)	43 (53.8)	7 (46.7)
Widowed	22 (23.2)	17 (21.2)	5 (33.3)
Single/divorced/separated	23 (24.2)	20 (25)	3 (20)

Average adherence in the supervised 12-week program was 86%. The most frequently reported reason for absence from classes was due to illness or medical problems (37.6% of the occasions of absences). Only 6.9% of sessions were cancelled due to weather (hot or wet). During the 12-week program, 12 people (15%) reported pain or discomfort due to aggravation of pre-existing injury/condition, with 16 events (0.95% of all sessions) reported. Five people (6.25%) missed exercise sessions due to aggravation of pre-existing injury/condition with a total of 15 sessions missed (0.89% of all sessions). One fall occurred during the exercise program with no severe injury. No serious adverse events occurred during the program.

**Fig. 3:**
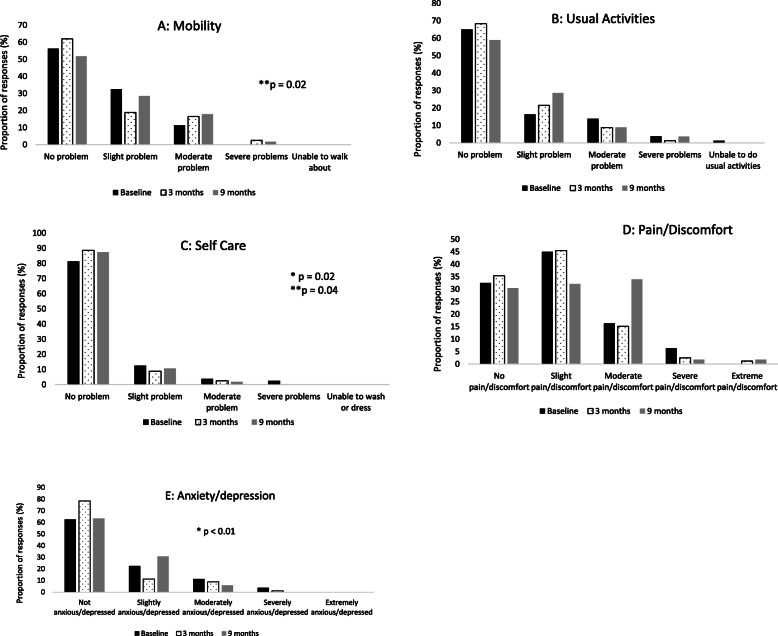
Proportion of responses by level of severity for EQ-5D-5L dimensions: Mobility (**a**), Usual Activities (**b**), Self-Care (**c**), Pain/Discomfort (**d**), Anxiety/depression (**e**) at baseline and at 3 and 9 months follow-ups. *Significant between baseline and 3 months. **Significant between baseline and 9 months

A significant increase in physical activity level was demonstrated following the intervention (CHAMPS caloric expenditure, frequency per week and total time in all exercise and in moderate exercise per week, *p* < 0.01, moderate to large effect sizes). Significant improvements were also demonstrated in all physical function measures (*p* < 0.01, small to large effect sizes), self rated quality of life (*p* = 0.04, small effect size), wellbeing (*p* < 0.01, small effect size), fear of falls (*p* < 0.01, medium effect size), falls risk (*p* < 0.01, medium effect size), depressive symptoms (*p* = 0.01, small effect size) and loneliness (*p* = 0.03, small effect size). No significant changes were demonstrated in socialisation and self-efficacy for exercise outcomes (p > 0.05) (Table 2). Changes in the EQ-5D-5L dimensions are presented in Fig. 3 with improvements shown in self-care (*p* <0.01) and depression (*p* = 0.02) domains.

**Table 2:** Primary and secondary outcome measures at baseline 3 and 9 months post intervention (mean ± SD)

Measure	Baseline	3 months	*P* value Baseline vs 3 months post (*n* = 80)	${\eta}_p^2$ Baseline vs 3 months	9 months	*P* value Baseline vs 9 months post (*n* = 58)	${\eta}_p^2$ Baseline vs 9 months	*P* value 3 months vs 9 months (*n* = 58)	${\eta}_p^2$ 3 months vs 9 months
Physical activity level CHAMPS (per week)									
All exercise - caloric expenditure	2581.9 ± 1705.6	3472.3 ± 2182.7	< 0.01*	0.05^d^	3272.1 ± 2078.5	0.01*	0.09^c^	0.6	0.00^d^
All exercise – frequency	15.6 ± 7.5	21.6 ± 8.6	< 0.01*	0.12^c^	20.16 ± 9.7	< 0.01*	0.2^b^	0.8	0.00^d^
All exercise – minutes	726.0 ± 416.8	915.5 ± 492.0	< 0.01*	0.04^d^	894.1 ± 519.9	0.03*	0.08^c^	0.5	0.00^d^
Moderate exercise - caloric expenditure	587.2 ± 914.8	1036.2 ± 1328.1	0.01*	0.03^d^	1091.0 ± 1379.4	0.02*	0.09^c^	0.2	0.02^d^
Moderate exercise - frequency	2.8 ± 3.4	5.6 ± 3.8	< 0.01*	0.12^c^	5.4 ± 5.7	< 0.01*	0.1^c^	0.7	0.00^d^
Moderate exercise - minutes	119.4 ± 178.6	197.4 ± 235.9	0.02*	0.03^d^	227.4 ± 290.5	0.01*	0.1^c^	0.07	0.05^d^
Physical function									
Sit to stand	10.1 ± 3.6	12.1 ± 4.1	< 0.01*	0.06^c^	12.5 ± 3.7	< 0.01*	0.3^b^	0.5	0.00^d^
Two-minute walk (m)	121.9 ± 30.4	133.1 ± 36.3	0.03*	0.02^d^	134.5 ± 32.6	< 0.01*	0.6^b^	0.4	0.01^d^
Walking speed (m/s)	1.3 ± 0.4	1.5 ± 0.4	< 0.01*	0.04^d^	1.6 ± 0.3	< 0.01*	0.3^b^	0.08	0.06^d^
Step test^a^	20.4 ± 6.7	27.3 ± 7.0	< 0.01*	0.20^b^	30.2 ± 8.0	< 0.01*	0.2^b^	^0.01*	0.12^c^
Health related quality of life									
Falls risk (FROP-Com)	11.0 ± 5.0	7.7 ± 3.9	p < 0.01*	0.09^c^	8.3 ± 4.5	< 0.01*	0.3^b^	ɬ0.01*	0.10^c^
Quality of life (EQ-5D-5L -VAS)	75.6 ± 14.8	80.1 ± 14.0	0.04*	0.02 ^d^	76.5 ± 16.3	0.5	0.00^d^	0.2	0.02^d^
Wellbeing (WHO-5)	61.8 ± 19.6	70.0 ± 17.5	< 0.01*	0.03^d^	66.6 ± 19.3	0.1	0.04^d^	0.1	0.03^d^
Loneliness UCLA 3	4.7 ± 1.6	4.3 ± 1.5	0.04*	0.01^d^	4.5 ± 1.5	0.9	0.00^d^	0.5	0.00^d^
Depression (GDS-15)	3.1 ± 2.9	2.5 ± 2.4	< 0.01*	0.01^d^	2.6 ± 2.5	0.02*	0.09^c^	0.8	0.00^d^
Fear of falls (Short FES-I)	11.1 ± 3.4	9.1 ± 2.2	< 0.01*	0.10^c^	9.5 ± 2.4	< 0.01*	0.2^b^	0.5	0.00^d^
Social isolation (LSNS6)	17.2 ± 5.4	17.6 ± 5.0	0.3	0.00^d^	17.5 ± 6.1	0.6	0.00^d^	0.7	0.00^d^
Self-Efficacy for Exercise (SEE)	57.9 ± 18.8	58.9 ± 17.3	0.5	0.00^d^	57.9 ± 16.3	0.8	0.00^d^	0.6	0.04^d^
Enjoyment (PACES)	43.4 ± 10.1	48.5 ± 7.2	< 0.01*	0.07^c^	45.8 ± 1.6	0.6	0.00^d^	< ¥0.01*	0.15^c^

*Significant at *p *< 0.05, ^improvement in Step Test; ɬ increase in falls risk (FROP-Com), ¥ reduction of enjoyment (PACES)

^a^The score is the sum of both limbs

^b^Large effect size

^c^Medium effect size

^d^Small effect size

For the 9 month follow up (*n* = 58) significant improvements from baseline were demonstrated in the frequency, duration and caloric expenditure of moderate physical activity and all type of exercises (*p* < 0.05, moderate to large effects size, Table 2). Significant improvements in all physical function measures were demonstrated between baseline and 9 months follow up (*p* < 0.05, moderate to large effect size, Table 2). Significant changes were observed only in the health related quality of life mobility and self-care domains (*p* < 0.05, EQ-5D-5L dimensions, Fig. 3). No changes were observed in the other health related quality of life measures apart from significant reductions in both fear of falls and falls risk (*p* < 0.01, Table 2). Very few changes were observed between 3 and 9 month follow ups as indicated in Table 2.

## Discussion

Participation in physical activity is important for the reduction and management of chronic diseases as well as to help older people remain mobile and independent. Only a quarter of older people meet the recommended national guidelines for physical activity [43]. Participation in the ENJOY Seniors Exercise Park program resulted in increases in physical activity level as well as physical and mental health benefits in the short term with sustained physical function benefit in the longer term.

Although there is strong evidence from randomized controlled trials to support the importance of physical activity, older people have low participation rates in physical activity programs aimed at achieving a variety of positive health outcomes, including falls prevention [44, 45]. Those who do commence a physical activity program as part of a research study often return to their inactive lifestyle behaviour once the study is completed, indicating that interventions that are not easy to apply in “real world” situations often do not sustain participation beyond the trial period [9]. Hence translating these studies into effective and sustained behaviour change remains a challenge [46]. Participation in the ENJOY project resulted in significant improvement in physical activity level (all CHAMPS outcomes) after the 12 weeks intervention and these improvements were sustained 6 months later (with no decline between 3 and 9 months), suggesting that participants remained physically active over the longer term. Importantly, the increase in the moderate exercise type (frequency and duration) indicates that participants exceeded the recommended physical activity participation of 150 min per week of moderate intensity. The sustained engagement in physical activity provides promising results for the potential benefits of scaling up such a program in order to achieve public health benefit for older people in the community.

Sustained participation in physical activity at a level to maintain or improve health by older people remains challenging to achieve, with fewer than half of older adults being active enough to achieve most of these health benefits [7]. Having national and international guidelines or recommendations appear insufficient to achieve this activity [47]. Providing widely available and accessible avenues for physical activity options, such as widespread implementation of outdoors Seniors Exercise Parks may assist improving participation levels. State and / or national policies supporting increased physical activity participation by older people may also be beneficial.

Loneliness and social isolation are greatest among older people and can pose significant physical and mental health risks [48, 49]. Loneliness, in addition to other physical and mental problems, gives rise to feelings of depression in older people [50]. Physical activity is one possible health promotion strategy that has positive effects on mental health in later life [51]. Improvement in depressive symptoms (as reported by the Depression domain in the Quality of Life scale and the Geriatric Depression Scale) and wellbeing were seen after the 3 months intervention. Slight improvement in loneliness (relational connectedness, social connectedness and self-perceived isolation) was demonstrated but with no changes in social isolation or social support. Caution must be taken, however, in the interpretation of the results as the values reported in both scales (UCLA3 and LSNS6) did not suggest that the participants experienced severe loneliness or lack of social engagement at baseline. The physical activity program incorporated group setting exercise activity followed by a social morning tea, which facilitated social connection. Enjoyment in physical activity was also significantly improved following the intervention. Given that fun, enjoyment and social interaction are key motivators for older people to take part in physical activity [52], these aspects should be an integral part of physical activity programs for older people. Consequently, the results highlight the beneficial effect of the Seniors Exercise Park program on general wellbeing.

We have previously demonstrated in a small randomized controlled trial the physical and social benefits of utilizing the Seniors Exercise Park program for older people [15, 16]. Pre-post studies such as the ENJOY trial may be considered to be less rigorous than randomised controlled trials, although in implementation research, especially where one or more previous randomised trials have demonstrated the approach to be effective (as in this study), this is considered acceptable [53]. The ENJOY project provides further evidence for the potential effectiveness of the physical and social activity program to improve quality of life and wellbeing on a larger scale beyond the 12 weeks supervised program. The approach utilized in the ENJOY project encompasses partnership with local governments to create an innovative enjoyable physical activity for older people with the utilization of specialized outdoor equipment. The availability of the equipment in community settings provides an advantageous set up where participants can have free access beyond the research trial. Moreover, the location in outdoor settings also offers additional benefits, as exercising outdoors is known for its’ health benefits for mental wellbeing [54]. Combining exercise, nature and social components may play a key role in engaging older people in physical activity and health promotion initiatives longer term.

Older people are at high risk of falls. Exercise programs have been shown to be effective in preventing falls in community-dwelling older people [55]. Balance and strengthening exercises in particular are important to be incorporated into exercise programs to reduce falls [55]. The Seniors Exercise Park program integrates multimodal exercise stations that target balance (unstable/uneven surfaces), strength and functional movements. This offers an important combination of different physiological aspects to obtain broader health benefits in addition to falls prevention, and is different to what is available in most traditional outdoor exercise equipment, which is usually focused on either cardiorespiratory or strength training [56]. The current sample population included people with high risk of falls (52.5% had previous falls) as well as high prevalence of complex medical issues. The sustained improvements in the physical function measures as well as the reduction in fear of falls and falls risk at 9 months suggest potential benefits to reduce the risk of falls.

This study has several limitations. Firstly, the spread of COVID-19 in early 2020 and associated restrictions and lockdown prevented older people in the community to be physically active using the Seniors Exercise Park in the latter stages of this project. This has adversely impacted on the ENJOY project data collection as participants were unable to access the Seniors Exercise Park sites as well as their ability to maintain their physical activity using this exercise modality. As such the data for the 9 months follow up included a smaller sample which could have potentially impacted on the results, leading to underestimation of the impact of the physical activity program on various health measures. The total dropout rate (*n* = 18, Fig. 1) of those who ceased participation in the project was 18.9% which is within the expected rate for exercise related interventional studies among older people [57, 58]. Secondly, the level of physical activity was measured using self-reported questionnaires which might not accurately reflect the actual physical activity level of the participants. However it is important to acknowledge that the CHAMPS questionnaire has been widely used in research and has been designed for use in evaluating interventions that primarily aim to increase levels of physical activity in older adults [22]. It is a reliable and valid questionnaire that is sensitive to change of the physical activity measures derived from it. The questionnaire was also tested and found suitable to be used with older Australians [59]. Finally, while our sample is representative of older Australians’ age, BMI and cultural background, it included a relatively high proportion of females with a small proportion of male participants. Although the risk and incidence of falls are greater in females, males are commonly under-represented in exercise intervention trials [60, 61]. Males have been reported to have specific preferences and characteristics of exercise interventions that are most likely to appeal to them [62]. The specific appeal or lack of appeal of this outdoor exercise park approach will need to be explored in future research.

## Conclusion

The results suggest that the Seniors Exercise Park may be an effective modality for improving older people’s physical function and wellbeing beyond an initial supervised program, and can be an important public health infrastructure investment in promoting physical activity for older people. Future work should focus on wider implementation of the program and on scaling up this initiative to achieve public health benefit for the community.

## Data Availability

The datasets generated and/or analysed during the current study are not publicly available due to ethical restrictions but are available from the corresponding author on reasonable request.

## Abbreviations

ENJOY: Exercise interveNtion outdoor proJect in the cOmmunitY for older people
RCT: Randomized controlled trial
TREND: Transparent Reporting of Evaluations with Nonrandomized Designs
CONSORT: Consolidated Standards Of Reporting Trials
AMTS: Abbreviated Mental Test Score
CHAMPS: Community Healthy Activities Model Program for Seniors
EQ-5D-5L: The 5-level EQ-5D
UCLA: University of California at Los Angeles Loneliness Scale
GDS-15: Geriatric Depression Scale
Short FES-I: Short Falls Efficacy Scale International
SEE: Self-Efficacy for Exercise
PACES: Physical Activity Enjoyment Scale
FROP-Com: The Falls Risk for Older People in the Community
SD: Standard deviation
LSNS6: 6 items Lubben Social Network Scale
WHO-5: World Health Organization Five wellbeing index
